# Effects of Gait Self-Efficacy and Lower-Extremity Physical Function on Dual-Task Performance in Older Adults

**DOI:** 10.1155/2017/8570960

**Published:** 2017-02-01

**Authors:** Diane K. Ehlers, Sarah E. Banducci, Ana M. Daugherty, Jason Fanning, Elizabeth A. Awick, Gwenndolyn C. Porter, Agnieszka Burzynska, Sa Shen, Arthur F. Kramer, Edward McAuley

**Affiliations:** ^1^University of Illinois at Urbana-Champaign, Urbana, IL, USA; ^2^Wake Forest University, Winston-Salem, NC, USA; ^3^University of Nebraska Medical Center, Omaha, NE, USA; ^4^Colorado State University, Fort Collins, CO, USA; ^5^Northeastern University, Boston, MA, USA

## Abstract

*Objectives.* Despite evidence of self-efficacy and physical function's influences on functional limitations in older adults, few studies have examined relationships in the context of complex, real-world tasks. The present study tested the roles of self-efficacy and physical function in predicting older adults' street-crossing performance in single- and dual-task simulations.* Methods.* Lower-extremity physical function, gait self-efficacy, and street-crossing success ratio were assessed in 195 older adults (60–79 years old) at baseline of a randomized exercise trial. During the street-crossing task, participants walked on a self-propelled treadmill in a virtual reality environment. Participants crossed the street without distraction (single-task trials) and conversed on a cell phone (dual-task trials). Structural equation modeling was used to test hypothesized associations independent of demographic and clinical covariates.* Results.* Street-crossing performance was better on single-task trials when compared with dual-task trials. Direct effects of self-efficacy and physical function on success ratio were observed in dual-task trials only. The total effect of self-efficacy was significant in both conditions. The indirect path through physical function was evident in the dual-task condition only.* Conclusion.* Physical function can predict older adults' performance on high fidelity simulations of complex, real-world tasks. Perceptions of function (i.e., self-efficacy) may play an even greater role. The trial is registered with United States National Institutes of Health ClinicalTrials.gov (ID: NCT01472744; Fit & Active Seniors Trial).

## 1. Introduction

Adults aged 65 years and older represent 8% of the population worldwide and are expected to comprise 16% by 2050. Rapid increases in the number of older adults by more than 250% are expected in many countries, driving the older adult population to nearly 1.5 billion worldwide [1]. Unfortunately, approximately 41% of older adults report at least one functional limitation related to activities of daily living, with 30% of older women and 19% of older men unable to perform one or more of the following physical functions: stooping or kneeling, reaching overhead, writing/grasping small objects, walking two to three blocks, or lifting ten pounds [2]. It is well-known these age-related declines in function can have significant implications for independence, quality of life, and safety [3, 4], in addition to increased risk of disability, morbidity, and mortality [5].

A strong body of evidence indicates that impaired physical function performance and self-efficacy may lead to an increase in perceived functional limitations [6–12] and subsequent disability [5]. Self-efficacy is a cognitive control system and refers to individuals' beliefs in their ability to carry out a specific course of action, particularly in challenging conditions [13]. As such, perceptions of capabilities, in addition to actual physical ability, are strongly related to individuals' actions [11, 14, 15]. In both cross-sectional and longitudinal models, McAuley and colleagues [6, 7] reported direct and indirect effects of self-efficacy on older women's functional limitations through physical function performance. Mullen and colleagues [12] replicated these findings in older adults using a way-finding task.

Despite considerable evidence in support of this pathway, a large amount of variability exists in the assessment of functional limitations [16]. Additionally, as functional limitations reflect individual perceptions, they may not fully capture the often complex nature of daily behavior and functional impairment [17–19]. Furthermore, few studies have examined the influences of self-efficacy and physical function performance in the context of complex, real-world tasks that may better reflect older adults' ability to carry out activities of daily living [20–22]. In an age where individuals face competing attentional demands (e.g., talking on a cell phone while walking or driving), older adults may be at even greater risk of compromised function and disability. It is imperative to test existing models using measures that may better gauge older adults' ability to independently carry out both simple and complex daily tasks.

A number of studies in the cognitive aging literature have utilized a naturalistic street-crossing simulation to describe older adults' real-world task performance under single- and dual-task conditions (i.e., crossing a virtual street without distraction versus crossing it while talking on a cell phone). Findings from these studies have mainly been descriptive and indicate that when compared with younger adults, older adults demonstrate slower gait speeds and face disproportionate performance costs with increased task difficulty and are more susceptible to performance costs under less demanding conditions [23]. Nagamatsu et al. [24] reported these performance declines may be even greater among older adults at risk of falls. These studies have suggested that when stimuli associated with concurrent tasks are similar, cognitive control demands and performance impairments are likely to be greater [23]. As such, higher levels of self-efficacy and physical function may serve to mitigate the impact of dual-task demands on task performance in older adults.

**Figure 1:**
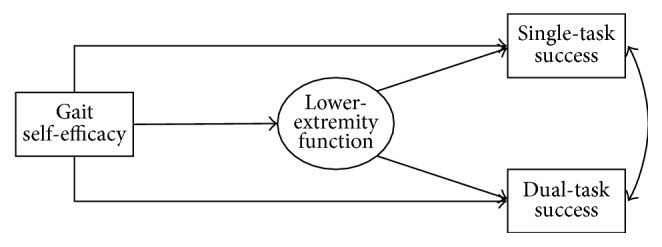
Hypothesized associations among gait self-efficacy, lower-extremity function, and task performance.

The purpose of this study was to determine the direct effects of lower-extremity physical function performance and the direct and indirect effects of gait self-efficacy, through functional performance, on older adults' street-crossing performance under single- and dual-task conditions (crossing a virtual street without distraction versus crossing it while talking on a phone) (Figure 1). We hypothesized that (a) greater gait self-efficacy and better performance on laboratory measures of lower-extremity physical function would directly predict better performance on the street-crossing task in both conditions; (b) self-efficacy would indirectly predict street-crossing performance through physical function in both conditions; and (c) the strength of the effects would be greater in the dual-task when compared with the single-task condition. We hypothesized these relationships would be evident independent of covariates known to be associated with self-efficacy, physical function, and/or dual-task performance, including age, cardiorespiratory fitness (CRF), and body mass index (BMI).

## 2. Methods

### 2.1. Participants

Findings of the present study represent secondary data collected as part of a randomized controlled exercise trial testing the effects of aerobic exercise training and aerobic exercise training + cognitive training on cognitive function and brain health in older adults. Participants in the present study were 195 older adults assessed at baseline of the trial. Eligible individuals were aged 60–79 years, English speaking, right-handed, local to the study location, willing to be randomized, not involved in another physical activity program, low-active (participated in 30+ minutes of moderate physical activity on no more than 2 days per week over the past 6 months), and free from neurological disorders and had no history of stroke or transient ischemic attack. The Mini-Mental State Exam (MMSE) [25] and Telephone Interview of Cognitive Status-Modified (TICS-M) [26] questionnaire were used to assess cognitive status. Individuals were eligible if they scored ≥27 on the MMSE [23] and >21 on the TICS-M. The project coordinator assessed all individuals for eligibility during a prescreening interview. This study was approved by the appropriate Institutional Review Board (IRB), and all participants signed the IRB-approved informed consent prior to participation in the study.

### 2.2. Measures

#### 2.2.1. Lower-Extremity Physical Function

Four measures assessing lower-extremity mobility, endurance, and strength were used to measure physical function. The battery included ascent/descent of a flight of 15 stairs, the timed 8-foot Up-and-Go, and chair stands [27, 28]. During the stair climb test, two scorers timed participants walking down followed by walking up a flight of stairs. Times were averaged to calculate separate scores for the ascent and descent portions of the test. During the timed 8-foot Up-and-Go, participants completed two trials starting seated in a chair, walking eight feet around a cone, and returning to seated position in the chair. The average of the two trials was used. Faster times on all three measures represented greater physical function performance. During the chair stand test, participants started in a seated position in a chair with their arms crossed against their chest, rose to a full stand without assistance, and returned to fully seated position in the chair as many times as they could within a 30-second interval. Completion of a greater number of chair stands indicated greater physical function performance. Chair stands score was reflected prior to data analysis to align its directionality relative to performance with the other three function measures.

#### 2.2.2. Self-Efficacy

Gait self-efficacy was measured using the Gait Self-Efficacy Scale (GES) [29]. The GES is a 6-item scale (*α* = 0.88) assessing individuals' beliefs in their ability to successfully walk despite obstacles, such as stairs or objects in one's path. Each item is scored on a scale of 0%, not confident at all, to 100%, highly confident in carrying out activities. Items were averaged to calculate an overall scale score.

#### 2.2.3. Street-Crossing Performance

A virtual street-crossing task conducted in the CAVE Automatic Virtual Environment (CAVE, Beckman Institute, Urbana, IL) was used to measure functional task performance. The CAVE street-crossing task is a virtual reality environment instantiation of multitasking. For a detailed description of the CAVE street-crossing task, see [23, 24, 30]. Briefly, participants walked on a self-propelled treadmill and were instructed to safely cross a virtual street synchronized with the treadmill. The street included two lanes of cars traveling 33 miles per hour at an intervehicle distance (IVD) of 75 or 90 meters (m), was free of extraneous obstacles, and did not include any safe zones where participants could wait for cars to pass [23, 24, 30]. Forty trials were conducted across four combinations (distraction [single-task or dual-task] × IVD [75 m or 90 m]). During dual-task trials, participants conversed with a researcher using a hands-free cell phone. Trials were conducted using a blocked design of ten trials per block. Each block consisted of single- or dual-task trials only but included 75 m and 90 m IVD trials (five each) randomly ordered within the block. The order of the blocks was counterbalanced across participants to mitigate the influence of learning effects. Participants were allowed 90 seconds to complete each trial before it was considered “timed out.” A trial was considered successful if the participant crossed the street without experiencing a collision or before the trial timed out. For the present analyses, street-crossing performance was defined as single- and dual-task success ratio, calculated as the number of successful trials divided by the total number of trials (*n* = 20 per condition).

#### 2.2.4. Covariates

Hypothesized covariates included age, CRF, and BMI. To determine CRF, participants completed a modified Balke graded maximal exercise test [31, 32]. Oxygen consumption was calculated from expired air sampled at 30-second intervals until the test was terminated volitionally by participants or by the supervising physician due to symptom limitation. CRF was defined as the highest recorded VO_2_ value. To calculate BMI, height and weight were measured (Seca stadiometer, Hamburg, Germany) prior to the grade exercise test.

### 2.3. Analysis

Data from the 40 street-crossing trials were reduced to two repeated measures representing single-task (i.e., no distraction) and dual-task (i.e., phone) success ratio. Preliminary analyses were conducted to examine the distribution of independent and dependent variables; identify outliers; describe differences in street-crossing performance between conditions; and measure the degree of association among covariates (age, CRF, and BMI), physical function (stair ascent and descent, 8-ft Up-and-Go, and chair stands), gait self-efficacy, and street-crossing success ratio. Due to extreme outliers, gait self-efficacy and street-crossing variables were winsorized at 3 standard deviations from the mean.

The following hypothesized effects were tested in a structural equation model: (a) direct paths from self-efficacy and physical function to single- and dual-task success ratio; (b) indirect paths from self-efficacy and covariates to success ratio through physical function; and (c) differences in the magnitude of these effects between single- and dual-task conditions. Several indices were used to assess model goodness-of-fit [33]: nonsignificant normal theory weighted chi-square statistic (*p* < 0.05) [34], root mean square error of approximation (RMSEA ≤ 0.05), standardized root mean residual (SRMR ≤ 0.05), and comparative fit index (CFI ≥ 0.90). Correlations with covariates and hypothesized directional effects were tested and (if not significant) were constrained to produce a final model best representing the data. To guard against bias, the final model was compared to possible alternate models that included street-crossing performance regressed on CRF and another that constrained this path. These comparisons were made with nested fit indices, including the Akaike Information Criterion (AIC) and sample-sized adjusted Bayesian Information Criterion (BIC), with smaller values indicating better goodness-of-fit.

**Figure 2:**
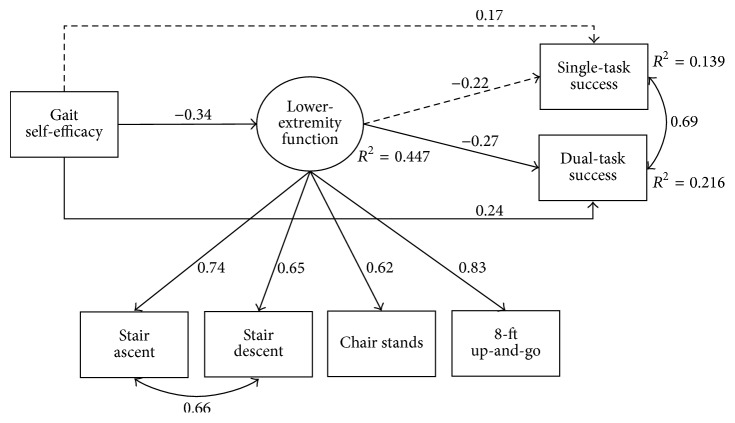
Structural equation model of relationships among gait self-efficacy, lower-extremity function, and street-crossing performance. Note: all coefficients represent standardized estimates from model output. Solid lines reflect statistical significance at *p* < 0.05, two-tailed. Covariate paths have been omitted for clarity but are presented in the text and in Supplemental Figure  1.

All variables were modeled as manifest constructs, except for a latent composite of lower-extremity physical function, comprised of stair ascent and decent, 8-ft Up-and-Go, and chair stands. Negative coefficients along pathways including the physical function latent composite represented positive associations. To avoid bias from the smaller sample size, effects were bootstrapped (5000 iterations of the entire sample) to estimate biased-corrected 95% confidence intervals (CIs) of the unstandardized direct, indirect, and total effects [35]. The single- and dual-task conditions were included simultaneously in the model as correlated outcomes. To assess the third hypothesis related to the increased magnitude of effect in the dual-task condition, we examined differences in bootstrapped corrected 95% CIs between the single- and dual-task effects. Results were considered significant at *p* < 0.05. The full-information maximum likelihood estimation was applied to account for missing data. Preliminary analyses were conducted in SPSS 22 (IBM, Armonk, NY) and structural equation modeling in MPlus 7.31 (Muthén & Muthén, Los Angeles, CA). To assist with interpretation of model paths in Figure 2, covariate paths were excluded and only standardized parameter estimates were presented. The full model is available in Supplemental Figure  1, in Supplementary Material available online at https://doi.org/10.1155/2017/8570960.

## 3. Results

### 3.1. Descriptive Measures

The sample (*N* = 195) is described in Table 1. Briefly, most participants were Caucasian and female and ranged in age from 60 to 78 years (M age = 65.31 ± 4.45 years). Significant differences in street-crossing success were observed such that success ratio was higher in single-task trials when compared with dual-task trials, *t*(194) = 4.54, *p* < 0.001. Correlations (Pearson's* r*) among variables tested in subsequent models are shown in Table 2.

### 3.2. Hypothesized Model

The latent lower-extremity function measurement model provided excellent fit to the data (*χ*^2^(1) = 0.23, *p* = 0.63; RMSEA = 0.00 [90% CI = 0.00 to 0.149]; SRMR = 0.004; CFI = 1.00), and all factor loadings were significant (*p* < 0.001). Next, we tested the direct effects of gait self-efficacy and lower-extremity function on single- and dual-task success ratio and the indirect effects of gait self-efficacy and covariates on single- and dual-task success ratio via lower-extremity function. The model, including indirect paths of gait self-efficacy and covariates through lower-extremity function, in addition to correlations among all covariates and with gait self-efficacy displayed adequate fit to the data (*χ*^2^(26) = 40.57, *p* = 0.03; RMSEA = 0.056 [90% CI = 0.02/0.09]; SRMR = 0.049; CFI = 0.977). However, examination of the model specification indicated that goodness-of-fit was improved by removing the effect of age, which in this restricted sample was not significantly related to lower-extremity function (*β* = 0.12, *p* = 0.10), and by adding correlations between CRF and success ratio outcomes (*χ*^2^(18) = 27.51, *p* = 0.07; RMSEA = 0.05 [90% CI = 0.00/0.09]; SRMR = 0.04; CFI = 0.986).

Standardized parameter estimates from the hypothesized model are presented in Figure 2 and Supplemental Figure  1. As hypothesized, gait self-efficacy (*β* = −0.34, *p* = 0.001), CRF (*β* = −0.40, *p* < 0.001), and BMI (*β* = 0.18, *p* = 0.03) were associated with lower-extremity function. Gait self-efficacy and lower-extremity function were significantly associated with street-crossing success in the dual-task condition (*β* = 0.24, *p* = 0.01; *β* = −0.27, *p* = 0.007, resp.) and marginally associated in the single-task condition (*β* = 0.17, *p* = 0.09; *β* = −0.22, *p* = 0.058, resp.). Taking into account the effects shared with covariates, the total effect of gait self-efficacy on street-crossing success was significant in both conditions (single-task: *β* = 0.25, *B* = 0.004, and *p* = 0.003; dual-task: *β* = 0.33, *B* = 0.007, and *p* < 0.001). Further, lower-extremity function partially accounted for the effect of gait self-efficacy on street-crossing success, which achieved statistical significance in the dual-task condition only (single: *β* = 0.07, *B* = 0.001, and *p* = 0.12; dual: *β* = 0.09, *B* = 0.002, and *p* = 0.04). Lower-extremity function accounted for 30% of the standardized effect of gait self-efficacy on single-task success and 28% of the effect on dual-task success. We also hypothesized that lower-extremity function may partially explain the effects of CRF and BMI on street-crossing success. While CRF and BMI were unrelated to single-task success ratio (indirect effects *p* = 0.08 and 0.18, resp.), in the dual-task condition, greater CRF was associated with greater street-crossing success via lower-extremity function (*β* = 0.11, *B* = 0.004, and *p* = 0.02). Although differences in statistical significance of this effect were observed between the two conditions, the magnitude of the effects was similar between conditions based on the overlap of the bias-corrected bootstrap 95% CIs of the unstandardized effects between single- and dual-task conditions (Table 3).

Because CRF proved to be a correlate of performance in single- (0.18, *p* = 0.005) and dual-task conditions (0.12, *p* = 0.09), as well as of gait self-efficacy (0.21, *p* = 0.001), we assessed the possibility that it alone accounted for performance better than the hypothesized effects. Goodness-of-fit was comparable between the hypothesized model that included a correlation between CRF and the outcome measures (AIC: 4855.98, adjusted BIC: 4859.76) and an alternate model including a direct effect of CRF (AIC: 4852.97, adjusted BIC: 4856.76; *χ*^2^(18) = 24.51, *p* = 0.14; RMSEA = 0.04 [90% CI = 0.00/0.08]; SRMR = 0.041; CFI = 0.99). Thus the hypothesized final model was supported as a viable description of the data. In the alternate model, CRF was directly associated with success ratio in single-task trials, but not in dual-task trials (single: *β* = 0.25, *B* = 0.008, and *p* = 0.001; dual: *β* = 0.16, *B* = 0.006, and *p* = 0.051). Further, the direct effects of gait self-efficacy and lower-extremity function on dual-task success ratio observed in the hypothesized model remained significant in the alternate model. Additionally, multiple goodness-of-fit indices were relatively worse in the model in which CRF was unrelated to success ratio (*χ*^2^(20) = 33.42, *p* = 0.03; RMSEA = 0.06 [90% CI = 0.02/0.09]; SRMR = 0.047; CFI = 0.980; AIC: 4857.88, adjusted BIC: 4861.46 versus hypothesized model: *χ*^2^(18) = 27.51, *p* = 0.07; RMSEA = 0.05 [90% CI = 0.00/0.09]; SRMR = 0.04; CFI = 0.986), indicating this meaningful covariate should be accounted for in addition to gait self-efficacy and lower-extremity function.

Finally, while age was not predictive of lower-extremity function, it was associated with street-crossing performance in both conditions. Therefore, an alternate model assessing the direct path from age to street-crossing success was tested. Goodness-of-fit in the alternate model including age (AIC: 5962.03, adjusted BIC: 5966.56; *χ*^2^(22) = 27.09, *p* = 0.21; RMSEA = 0.03 [90% CI = 0.00/0.07]; SRMR=0.039; CFI = 0.99) was worse when compared with the hypothesized model. Age was associated with street-crossing success in both single- and dual-task trials (*β* = −0.018, *p* = 0.01; *β* = −0.18, *p* = 0.01, resp.). Despite this, all hypothesized effects reported above were observed independent of this direct effect of age. Although age may be a meaningful covariate of street-crossing performance, its contribution to behavioral differences among older adults sampled from this narrow age range was small and its inclusion compromised the parsimony of the model. Therefore, the hypothesized model was retained.

## 4. Discussion

This study is one of the first to explore relationships among self-efficacy, physical function, and older adults' performance on a real-world, potentially risky everyday behavior—street-crossing while talking on a phone. Consistent with similar studies, crossing success was significantly lower in dual-task trials [23, 24]. Greater gait self-efficacy and lower-extremity physical function were predictive of greater street-crossing success, particularly in the dual-task condition. The indirect effect of self-efficacy on dual-task success provides further evidence that self-efficacy is an important behavioral determinant independent of physical function and fitness. These results extend previous models linking self-efficacy, physical function, and functional limitations in older adults and suggest more research in the context of complex, real-world tasks is needed.

Although street-crossing performance declined with increased task complexity, higher levels of lower-extremity physical function and self-efficacy attenuated these declines. These results are consistent with previous work by Hausdorff and colleagues [21] in which slower usual gait speed and 8-foot Up-and-Go time among healthy older adults were associated with greater dual-task gait decrements. A large body of evidence supports the role of preserved physical function in mitigating perceived functional limitations and maintaining quality of life into older adulthood [5–9]. Findings of the present study, in the context of a real-world functional task, add to this evidence base and warrant more studies employing naturalistic simulations of activities of daily living. The prevalence of attention demanding technologies continues to grow rapidly, and previous research indicates that even high functioning older adults do not prioritize gait and function during dual-task situations [21]. As such, the consequences for older adults' daily functioning, safety, and quality of life may be considerable. Behavioral and environmental interventions and supports that enhance functional performance represent priority health promotion strategies for older adults increasingly faced with a life space impinged by overlapping attentional stimuli.

Our findings relative to self-efficacy further underscore the need for additional examination of trajectories of functional impairment in complex, real-world settings. Similar to previous studies on perceived functional limitations [6, 7, 12, 15], self-efficacy emerged as a predictor of task performance. The significant indirect effect follows the social cognitive framework which posits that individuals with greater personal efficacy related to skills (e.g., gait) are more likely to engage in activities that develop competencies such as physical function performance [13]. Although we expected to observe direct and indirect effects in both conditions, it is not surprising the effects were evident in the dual-task condition only. Self-efficacy theory hypothesizes its predictive power is most potent under more demanding conditions, a proposition that has been consistently corroborated across populations and behaviors [13, 36]. The total effect of self-efficacy in predicting street-crossing success, in addition to the reduced role of physical function on the indirect effect of self-efficacy in dual-task trials, also suggests efficacy expectations may represent a stronger determinant of an individual's ability to carry out complex activities of daily living when compared with physical function and fitness. Giannouli and colleagues [22] found that laboratory measures of gait and function were not strong predictors of older adults' performance on real-world mobility tasks and argued that factors other than physical capacity (e.g., cognition, self-efficacy) play important roles in older adults' real-life mobility. Evidence from the behavioral literature further suggests that self-efficacy's role in determining behaviors may be even stronger than that of actual ability [11, 13–15]. This is important, as individuals who perceive a task as overwhelming or threatening may be less likely to engage in the activity even if individual capacity does not prohibit participation [13, 22]. Unfortunately, these behavioral decisions may lead to reduced physical activity, impaired mobility, and loss of independence [3, 4], all key factors to successful aging.

This has important implications for the design of behavioral and environmental interventions for older adults. While previous evidence indicates self-efficacy and physical function are important predictors of adults' quality of life throughout the aging process [4, 6, 7], the results of the present study indicate self-efficacy and physical function are high priority health promotion strategies amidst progressively more complex life spaces for older adults. Physical activity interventions have successfully utilized self-efficacy based approaches to improve physical function and quality of life in older adults [37, 38]. Clearly there is a need for additional research examining the pathway between changes in self-efficacy, physical function, and street-crossing performance after an exercise intervention. This may further illuminate valuable health promotion strategies for improving older adults' ability to carry out complex activities of daily living and remain functionally independent.

Findings from this study contribute to the literature on physical function and activities of daily living and present several directions for future research. Previous research indicates that gait in dual-task conditions may be multidimensional and rely upon various factors, including physical function, self-efficacy, executive function, and mental well-being [21, 22, 39, 40]. For example, some studies have proposed that changes in gait speed during dual-task street-crossing may be related to executive functioning and attentional control, as opposed to physical function [24, 41]. However, findings by Gothe and colleagues [39] suggest there may be an indirect effect of executive function on older adults' task performance through physical function. Further, McAuley and colleagues [42] observed executive function as a predictor of older adult's self-efficacy. Results of the present study also revealed fitness as an important predictor of task performance along with self-efficacy and physical function. The indirect effect of CRF on dual-task success was similar to that of self-efficacy, suggesting it may operate in concert with self-efficacy in determining task performance through physical function. A number of randomized controlled trials have also observed effects of CRF on brain health and cognitive functions [43–46]. How these findings translate to performance on activities of daily living is clinically important, especially as the prevalence of cognitively demanding technologies continues to grow. Research exploring interactions among self-efficacy, fitness, physical function, executive function, and other cognitive processes in the context of the street-crossing task may provide the information needed to develop strategies for improving older adults' functional capacities and quality of life.

Despite its strengths, this study is not without limitations. Although our sample was larger than other street-crossing studies, it was comprised of predominantly well-educated, high functioning, and Caucasian older adults (Table 1). The lack of significant findings in the single-task condition is not surprising, as these trials may not have been functionally challenging enough for our sample. Despite this, the effects observed in the dual-task condition indicate a protective role of self-efficacy and physical function among healthy aging adults. This is important, as even healthy, high functioning older adults suffer dual-task decrements [20, 23]; however, lower functioning and frail older adults may experience the greatest costs [24]. Additionally, surveillance data indicate that less educated individuals and racial/ethnic minorities suffer disproportionate physical and cognitive decline with aging [47, 48]. Future research with a more diverse sample is needed to draw further conclusions about relationships among self-efficacy, physical function, and complex task performance. The path analysis provided a robust test for this complex relation, but the analysis is impacted by the degree of unreliability of the measures employed [49], and the bias-corrected bootstrapping procedure can only partially address this limitation. Future study should consider collecting multiple indices of each construct to allow for complete latent modeling that can more directly remove the influence of measurement error from effect estimates. Further, the impacts of cognitive processes previously evidenced to be associated with street-crossing performance, such as executive function and attentional control, were not considered in the present analysis. Additional research examining the effects of both cognitive processes and functional capacity on street-crossing performance is needed.

We also acknowledge the cross-sectional nature of this study which precludes conclusions related to causal associations. Examination of longitudinal relationships among changes in self-efficacy, physical function performance, and street-crossing performance after an exercise intervention may provide richer information related to older adults' successful functioning in dual-task environments. Street-crossing task was a useful measure of performance on a real-world activity, and the CAVE environment provided only a simulation of street-crossing. As it would be unsafe and unethical to test an actual street-crossing scenario, the extent to which performance on the simulation transfers to actual street-crossing success is not known.

## 5. Conclusion

Our findings suggest self-efficacy and physical function play important roles in older adults' safe mobility, particularly amidst overlapping attentional stimuli. Additional research testing physical function's influence in concert with a number of cognitive processes, including self-efficacy and executive function, and physical fitness may elucidate information needed to design better programs and environmental supports that optimize safe mobility in a growing population of older adults.

## Supplementary Material

Supplementary Figure 1 is a more comprehensive version of Figure 2 and illustrates relationships and structural paths among covariates (i.e., body mass index, cardiorespiratory fitness), in addition to gait self-efficacy, lower-extremity function, and street crossing success.

## Figures and Tables

**Table 1:** Sample characteristics.

	*N*	(%)
	M	±SD
Age (years)	65.31	±4.45
Female	122	(62.6)
Body mass index (kg/m^2^)	30.93	±5.28
Race		
Caucasian	167	(85.6)
African American	23	(11.8)
Asian	5	(2.6)
Education		
Noncollege graduate	78	(40.0)
College graduate	117	(60.0)
Income^a^		
≤$40,000	47	(28.8)
>$40,000	116	(71.2)
Marital status		
Married	118	(60.5)
Partnered	6	(3.1)
Single	24	(12.3)
Divorced/separated	26	(13.3)
Widowed	21	(10.8)
Cardiorespiratory fitness (mL/kg·min)	19.90	±4.54
Stair ascent (sec)	7.85	±1.88
Stair descent (sec)	7.40	±2.42
8-ft Up-and-Go (sec)	5.94	±1.16
Chair stands (total *n*)	11.34	±2.53
Gait self-efficacy (%)^b^	94.76	±10.38
Single-task success ratio (%)	79.87	±16.16
Dual-task success ratio (%)	76.12	±17.76

M = mean; SD = standard deviation.

^a^ *n* = 163; 32 participants chose not to answer.

^b^ *n* = 180.

**Table 2:** Bivariate correlations among constructs.

	1	2	3	4	5	6	7	8	9	10
(1) Age	—	−0.24^*∗*^	−0.08	−0.28^*∗∗*^	0.21^*∗∗*^	0.18^*∗*^	0.22^*∗∗*^	−0.16^*∗*^	−0.24^*∗∗*^	−0.27^*∗∗*^
(2) CRF	—	—	−0.42^*∗∗*^	0.22^*∗∗*^	0.36^*∗∗*^	0.33^*∗∗*^	−0.46^*∗∗*^	0.39^*∗∗*^	0.37^*∗∗*^	0.31^*∗∗*^
(3) BMI	—	—	—	−0.21^*∗∗*^	0.32^*∗∗*^	0.32^*∗∗*^	0.35^*∗∗*^	−0.19^*∗∗*^	−0.23^*∗∗*^	−0.25^*∗∗*^
(4) Gait self-efficacy	—	—	—	—	−0.45^*∗∗*^	−0.42^*∗∗*^	−0.33^*∗∗*^	0.28^*∗∗*^	0.27^*∗∗*^	0.36^*∗∗*^
(5) Stair ascent	—	—	—	—	—	0.82^*∗∗*^	0.61^*∗∗*^	−0.42^*∗∗*^	−0.32^*∗∗*^	−0.35^*∗∗*^
(6) Stair descent	—	—	—	—	—	—	0.53^*∗∗*^	−0.35^*∗∗*^	−0.31^*∗∗*^	−0.32^*∗∗*^
(7) 8-ft Up-and-Go	—	—	—	—	—	—	—	−0.55^*∗∗*^	−0.28^*∗∗*^	−0.28^*∗∗*^
(8) Chair stands	—	—	—	—	—	—	—	—	0.12	0.16^*∗*^
(9) Single-task success ratio	—	—	—	—	—	—	—	—	—	0.73^*∗∗*^
(10) Dual-task success ratio	—	—	—	—	—	—	—	—	—	—

CRF = cardiorespiratory fitness; BMI = body mass index.

^*∗∗*^ *p* < 0.01; ^*∗*^*p* < 0.05.

**Table 3:** Confidence intervals for direct, indirect, and total effects of self-efficacy, physical function, and covariates on street-crossing success.

	Single-task success ratio						Dual-task success ratio					
	Bias-corrected bootstraped 95% CI						Bias-corrected bootstraped 95% CI					
	Direct		Indirect		Total		Direct		Indirect		Total	
	Lower	Upper	Lower	Upper	Lower	Upper	Lower	Upper	Lower	Upper	Lower	Upper
Gait self-efficacy	0.00^a^	0.006	0.00	0.003	0.002	0.007	0.002	0.008	0.001	0.004	0.004	0.009
Lower-extremity function	−0.08	−0.002	—	—	−0.08	−0.002	−0.10	−0.02	—	—	−0.10	−0.02
Covariates												
BMI	—	—	−0.003	0.00	−0.003	0.00	—	—	−0.004	0.00	−0.004	0.00
CRF	—	—	0.00	0.006	0.00	0.006	—	—	0.002	0.008	0.002	0.008

Notes: CI: confidence interval, BMI: body mass index, and CRF: cardiorespiratory fitness.

95% CIs that do not overlap with zero support an effect at *p* < 0.05.

^a^Unstandardized parameter estimates.
