# Supplementary material for: Effects of Gait Self-Efficacy and Lower-Extremity Physical Function on Dual-Task Performance in Older Adults
Source: Biomed Res Int. 2017 Feb 1;2017:8570960. doi: 10.1155/2017/8570960 (PMC5309416; doi:10.1155/2017/8570960)
Supplement: Supplementary file 1 — Supplementary Figure 1 is a more comprehensive version of Figure 2 and illustrates relationships and structural paths among covariates (i.e., body mass index, cardiorespiratory fitness), in addition to gait self-efficacy, lower-extremity function, and street crossing success. [file 8570960.f1.docx]

Supplemental Figure 1. Structural equation model of relationships among covariates, gait self-efficacy, lower-extremity function, and street-crossing performance

-.40

.18

Gait

Self-efficacy

Lower-extremity Function

Dual-task Success

Single-task Success

Body Mass Index

Cardiorespiratory Fitness

-.34

.18

-.40

.17

-.27

-.22

.24

.69

.12

-.22

.21

Note: All coefficients represent standardized estimates from model output. Solid lines reflect statistical significance at *p*<.05, two-tailed. Age was also tested as a correlate of exogenous predictors and as an indirect predictor of street-crossing performance, but, due to non-significant effects, is not represented in the model.
